# A Novel SETX Mutation in a Taiwanese Patient with Autosomal Recessive Cerebellar Ataxia Detected by Targeted Next-Generation Sequencing, and a Literature Review

**DOI:** 10.3390/brainsci12020173

**Published:** 2022-01-28

**Authors:** Ping-I Chiang, Ting-Wei Liao, Chiung-Mei Chen

**Affiliations:** 1 Center for Medical Education in English, Poznan University of Medical Sciences, 60-512 Poznan, Poland; 78429@student.ump.edu.pl; 2 Linkou Medical Center, Department of Neurology, Chang Gung Memorial Hospital, Taoyuan 33305, Taiwan; timmyibm2002@cgmh.org.tw; 3 Department of Neurology, College of Medicine, Chang Gung University, Taoyuan 33305, Taiwan

**Keywords:** oculomotor apraxia type 2, autosomal recessive spinocerebellar ataxia, SETX, novel mutation, next-generation sequencing

## Abstract

Ataxia with oculomotor apraxia type 2 (AOA2), also known as autosomal recessive spinocerebellar ataxia with axonal neuropathy-2 (SCAN2) (OMIM #606002), is a neurodegenerative disorder characterized by early-onset progressive cerebellar ataxia, polyneuropathy, and elevated levels of alpha-fetoprotein. It is caused by mutations in the SETX (OMIM #608465) gene. The prevalence of this disease is widely varied, from non-existent up to 1/150,000, depending on the region. Until now, no cases of AOA2/SCAN2 have been reported in Taiwan. Methods: Next-generation sequencing was used to detect disease-causing mutations of SETX in a Taiwanese patient presenting with autosomal recessive cerebellar ataxia, polyneuropathy, and elevated alpha-fetoprotein. The candidate mutations were further confirmed by polymerase chain reaction (PCR) and Sanger sequencing. Results: A compound heterozygous mutation of SETX c.6859C > T (p.R2287X) and c.7034-7036del was identified. The c.6859C > T (p.R2287X) has been previously found in a Saudi Arabia family, whereas c.7034-7036del is a novel mutation. Both mutations were predicted by bioinformatics programs to be likely pathogenic (having a damaging effect). We also reviewed the literature to address the reported clinical features of AOA2 from different populations. Conclusions: To our knowledge, we are the first to report a Taiwanese patient with AOA2/SCAN2, a result obtained by utilizing next-generation sequencing. The literature review shows that ataxia, polyneuropathy, and elevated AFP are common features and ocular motor apraxia (OMA) is a variable sign of AOA2 from different populations. OMA is rare and saccadic ocular pursuit and nystagmus are common in East Asian AOA2.

## 1. Introduction

Ataxia with oculomotor apraxia type 2 (AOA2)/autosomal recessive spinocerebellar ataxia with axonal neuropathy-2 (SCAN2) (OMIM #606002) is characterized by early-onset progressive cerebellar ataxia, polyneuropathy, and elevated levels of alpha-fetoprotein (AFP). It is caused by loss-of-function mutations in the gene SETX (OMIM #608465) on chromosome 9q34.1. SETX gene encodes for senataxin, a 2677-amino acid protein that is ubiquitously expressed. Senataxin contains a DNA/RNA helicase domain at its C-terminus, which shows homology to the helicase domain of the yeast protein Sen1p and an N-terminal domain that is important for protein–protein interaction [1]. Senataxin has been shown to play important roles in transcription regulation, protecting the integrity of the genome against oxidative and other forms of DNA damage [2], neuritogenesis [3], spermatogenesis [4], and autophagy [5]. Mutations in SETX are also associated with the phenotype of juvenile-onset Amyotrophic Lateral Sclerosis (ALS) [5,6]. AOA2/SCAN2 belongs to the group of neurological conditions known as Autosomal Recessive Cerebellar Ataxias (ARCAs), which currently comprises over 40 disorders. The pathology of ARCAs mainly involves degeneration of the cerebellum, brain stem, spinal cord, and/or peripheral nerves. Key features include cerebellar ataxia, pyramidal tract signs, peripheral sensorimotor neuropathy, and the absence or weak deep-tendon reflexes, with disease onset usually before 20 years of age [7]. ARCAs are not only composed of disorders with overlapping presentations but also heterogeneous phenotypes; hence, differentiation between the different types of ARCAs poses a great challenge for physicians. Luckily, with the guidance of algorithms and advances in genetic analysis, such as next-generation sequencing (NGS), diagnosis can be pinpointed, which greatly improves the accuracy in diagnosing these disorders.

Here, we describe the first case of AOA2/SCAN2 diagnosed in Taiwan, including the clinical features of the disease and the methods we used to identify the novel pathogenic variant found by NGS. In addition, we include a brief review of the literature on the relevant topics.

## 2. Subjects and Methods

### 2.1. Subjects

A female patient presenting with ARCA with polyneuropathy, her parents, and 100 normal controls were recruited from the Department of Neurology, Chang Gung Memorial Hospital, Taiwan. This study was performed after obtaining written informed consents from the participants.

### 2.2. Genetic Study

According to the patient’s clinical presentation and results of comprehensive examinations, although the autosomal dominant inheritance is not likely, triplet expansions in the genes that cause spinocerebellar ataxia type 1, 2, 3, 6, 7, 8, 12, and 17 were excluded first. AOA1, AOA2, Ataxia Telangiectasia (AT, AOA3), AOA4, Spinocerebellar Ataxia with Axonal Neuropathy (SCAN1), Ataxia Telangiectasia-Like Disease (ATLD), and Friedreich’s Ataxia (FRDA) were then considered as the differential diagnosis. Due to the overlap and heterogeneous spectrum of these diseases, NGS was performed to identify the disease-causing gene mutations. The candidate mutations were further confirmed by polymerase chain reaction (PCR) and Sanger sequencing. These mutations were also performed in the parents to examine the family co-segregation. Furthermore, 100 normal controls were examined for the identified mutations.

### 2.3. NGS and Bioinformatics

DNA was extracted from peripheral blood leukocytes according to standard protocols and purity was checked using a NanoPhotometer^®^ spectrophotometer (IMPLEN, Westlake Village, CA, USA). DNA concentration was measured using Qubit^®^ DNA Assay Kit in Qubit^®^ 2.0 Flurometer (Life Technologies, Carlsbad, CA, USA). Fragment distribution of DNA library was measured using a DNA Nano 6000 Assay Kit of the Agilent Bioanalyzer 2100 system (Agilent Technologies, Santa Clara, CA, USA). A total of 1.0 μg genomic DNA per sample was used as the input material for the DNA library preparation. Sequencing libraries were generated using an Agilent SureSelect Human All Exon kit (Agilent Technologies, Santa Clara, CA, USA) following the manufacturer’s recommendations, and index codes were added to each sample. Briefly, fragmentation was carried out by a hydrodynamic shearing system (Covaris, Woburn, MA, USA) to generate 180–280 bp fragments. Remaining overhangs were converted into blunt ends via exonuclease/polymerase activities and enzymes were removed. After adenylation of the 3′ ends of the DNA fragments, adapter oligonucleotides were ligated. DNA fragments with ligated adapter molecules on both ends were selectively enriched in a PCR reaction. After the PCR reaction, the library was hybridized with a liquid phase with a biotin-labeled probe, and magnetic beads with streptomycin were used to capture the 334,378 exons spanning 20,965 genes. Captured libraries were enriched in a PCR reaction to add index tags to prepare for hybridization. Products were purified using an AMPure XP system (Beckman Coulter, Beverly, MA, USA) and quantified using an Agilent high-sensitivity DNA assay on an Agilent Bioanalyzer 2100 system.

Software, the Burrows–Wheeler Aligner (BWA), Sequence Alignment/Map (Samtool), and Picard were used for alignment analysis; Genome Analysis Toolkit (GATK) for SNP/InDel detection; and ANNOVAR for annotation of the variants. The databases VarCards (http://varcards.biols.ac.cn) (accessed on 5 January 2022) and Mutation Taster were used to predict the potential structure and function of the protein affected by the identified mutations.

## 3. Results

### 3.1. Clinical Assessments

A Taiwanese 27-year-old woman, born to non-consanguineous parents with an unremarkable family history (no family history of ALS), started to have progressive gait disturbance since the age of 16. It started from difficulties in running, standing on one leg, or walking straight. Later, she became prone to falling and choking while drinking in the following years. Although there were no signs of cognitive impairment, academic performance was poor, and she dropped out of school at the age of 20.

Due to a progressive unsteady gait with increasing falling episodes, the patient was first evaluated for her condition at our hospital at the age of 20 (2011). Initial neurological examinations revealed saccadic ocular pursuit and gaze-evoked nystagmus without oculocutaneous telangiectasia or retinitis pigmentosa. Motor examinations showed normal muscle power in the upper limb, and mild weakness in the distal muscles of lower limbs without upper motor neuron signs. Sensory examinations showed no abnormalities. Deep tendon reflex (DTR) showed generalized areflexia. Positive cerebellar signs included ataxic gait and bilateral finger to nose dysmetria without evident scanning speech. Brain MRI showed mild cerebellar atrophy. The sensory-evoked potential (SEP), motor-evoked potential (MEP), visual-evoked potential (VEP), electroencephalogram (EEG), and brainstem auditory-evoked potential (BAEP) examinations were all normal. The first nerve conduction studies (NCS) showed absence of bilateral sural sensory nerve action potentials (SNAPs), and decreased amplitudes of bilateral median and ulnar SNAPs with a marginally slow motor nerve conduction velocity of the bilateral peroneal nerve, which suggest mainly axonal polyneuropathy with features of mild demyelinating polyneuropathy. Laboratory results showed elevated AFP 23.1 ng/mL (normal range < 15 ng/mL) and normal albumin of 4.61 g/dL (normal range: 3.5–5.5 g/dL), without hypercholesterolemia. Lipoprotein electrophoresis test and creatine kinase (CK), vitamin E, copper and ceruloplasmin levels were all normal. Cardiomegaly was not present on echocardiography.

**Figure 1:**
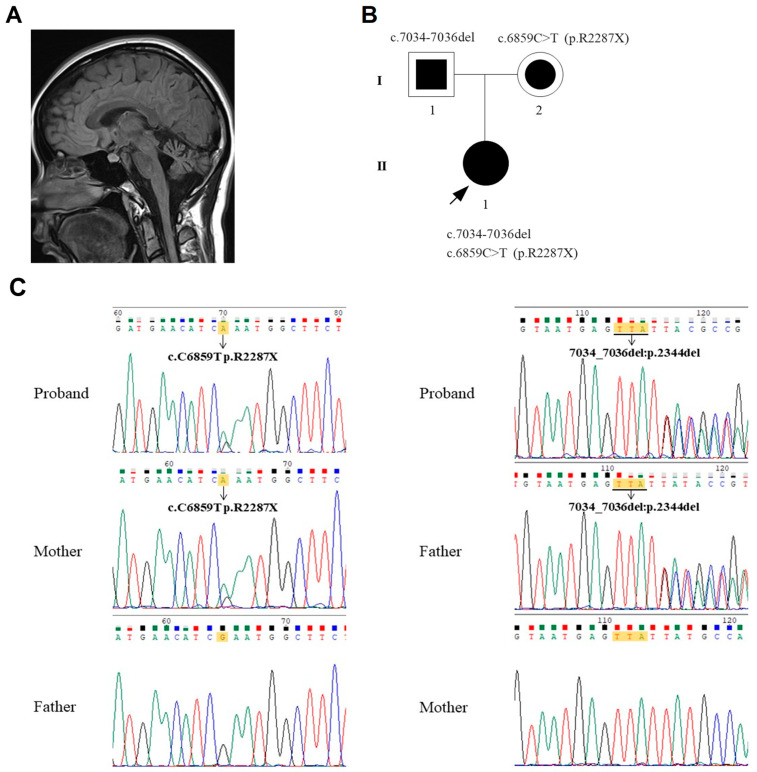
(**A**) Image of a brain MRI showing cerebellar and brain stem atrophy. (**B**) Pedigree of the patient’s family. The arrowhead with the closed square indicates the proband (II-1). Semi-closed square (I-1) and circle (I-2) indicate her asymptomatic father and mother, respectively. (**C**) Sequencing results showed the mutations c.6859C > T (p.R2287X) inherited from the proband’s mother (**left**) and c.7034_7036del inherited from the proband’s father (**right**).

Seven years after her first evaluation, at the age of 27, she came to our hospital for the 2nd evaluation. Most deficits in the previous evaluation deteriorated, but she was still ambulating with slight assistance. New clinical features included chorea in the right hand and mild pes cavus in the feet. There were no signs of dystonia or tremor of the head and arms. Brain stem atrophy in addition to cerebellar atrophy was shown on MRI (Figure 1A), and NCS this time showed axonal polyneuropathy with absence of bilateral sural SNAPs, absence of left peroneal compound muscle action potential (CMAP), and significantly decreased amplitudes of the right peroneal and bilateral tibial CMAPs. Her AFP levels increased from 23.1 to 30.5 ng/mL. 

### 3.2. Results of Genetic Testing

Analysis of the NGS results revealed a compound heterozygous mutation of the SETX gene: c.6859C > T (p.R2287X) in exon 22 and c.7034-7036del in exon 23. The nonsense mutation c.6859C > T (p.R2287X) has been previously reported in one AOA2 family in Saudi Arabia [8]. No likely pathogenic or pathogenic mutations were found in the following genes: APTX encoding for aprataxin (AOA1), ATM encoding for ATM serine/threonine kinase (AT), PNKP encoding for polynucleotide kinase 3’-phosphatase (AOA4), TDP1 encoding for tyrosyl-DNA phosphodiesterase 1 (SCAN1), and MRE11A encoding for meiotic recombination 11 (ATLD).

Sanger sequencing was performed on the SETX gene for the proband, her father, her mother, and 100 normal controls. The results showed that the proband inherited the c.6859C > T (p.R2287X) from her mother and c.7034-7036del from her father (Figure 1B,C). The Sanger sequencing also showed no mutation of c.6859C > T and c.7034-7036del in the 100 normal controls.

### 3.3. Structure and Function Prediction of the Mutations

The database VarCards (http://varcards.biols.ac.cn) (accessed on 5 January 2022) and Mutation Taster, respectively, was used to predict the potential structure and function of the c.6859C > T (p.R2287X) (Table 1) and c.7034-7036del (Table 2) mutations in the SETX gene.

The Mutation Taster predicts that c.6859C > T (p.R2287X) is disease_causing_automatic and phyloP suggests that this nucleotide is highly conserved (Table 1)**.** The Mutation Taster predicts that the c.7034-7036del mutation causes a change in the amino acid sequence, which may affect the protein feature and alter a splice site. The c.7034-7036del was neither found in ExAC nor 1000~G and was not found in the 100 normal controls. Both phyloP/phastCons indicate that the c.7034_7036 nucleotides and the amino acid at 2344 are highly conserved (Table 2). The results indicate that both mutations have a “damaging effect” and suggest they are likely pathogenic.

The nonsense mutation c.6859C > T (p.R2287X) is classified as “Pathogenic” according to American College of Medical Genetics and Genomics (ACMG) guidelines [9], as it fulfills the criteria (1) PVS1, (2) PM2, and (3) PP3. However, the c.7034-7036del mutation has not yet been reported in the literature or in the Human Gene Mutation Database (HGMD). This new mutation fulfills the criteria of being a “Likely pathogenic” variant according to the ACMG guidelines: (1) PM2, (2) PM4, (3) PP3, and (4) PP4. Family segregation studies documented a status of heterozygous carrier for each of the two mutations in the proband’s relatives (Figure 1C).

## 4. Discussion

### 4.1. Differential Diagnosis and Literature Review

Early-onset of cerebellar ataxia is a group of various hereditary disorders featured mainly by ataxia. Due to the significant overlap in clinical presentation, the diagnosis is usually challenging. Our patient presented with cerebellar ataxia, polyneuropathy, generalized areflexia, saccadic pursuit, slow saccadic eye movements without oculomotor apraxia (OMA), chorea, and elevated levels of AFP. After narrowing down the possible differential diagnoses according to algorithms and information available in the literature regarding ARCAs [7,10,11], our main differentials included AOA1, AOA2, AT (AOA3), AOA4, ATLD, and SCAN1, all of which are characterized by gait ataxia and polyneuropathy. The presence of elevated AFP is suggestive of AOA1, AOA2, AT (AOA3), and AOA4. Apart from specific signs seen in each disease category, AFP thresholds may also provide us guidance in distinguishing among the differentials [12,13]. AFP levels are significantly elevated in most of AT and AOA2 patients, whereas they may also be slightly higher in AOA1 and AOA4 than in normal individuals. It is worthy to note that, in some cases of AOA2, the AFP levels may be normal initially [14]; therefore, AFP levels assessments should be repeated during the course of an ataxia of unknown etiology. It should be noted that we did not check the patient’s IgA or IgG levels; however, they may be of diagnostic value in AT [15]. Other clues that favor the diagnosis of AOA2 in comparison to the other differentials include no ocular telangiectasis or immunodeficiency (to exclude AT and ATLD), teenage onset (AOA1, AOA4, and AT typically have an onset < 10 years), normal albumin (more commonly decreased in AOA1), and cholesterol levels (more commonly increased in AOA1) [13,16,17]. Despite the name, oculomotor apraxia (OMA) is not a cardinal trait in AOA2, and OMA was observed in approximately 50% or less in most reported series of AOA2 (Table 3).

The frequency of OMA is higher in AOA1 (86%), AOA4 (100%), and AT (100%) than in AOA2 [16]. Due to the above observation, Duquette et al. [18] emphasized that OMA is not a universal finding in AOA2 and suggested the name “spinocerebellar ataxia, autosomal recessive, with axonal neuropathy-2” (SCAN2) to distinguish it from SCAN1 (OMIM #607250), which is caused by a homozygous mutation in the TDP1 gene and characterized by cerebellar ataxia with atrophy, peripheral axonal sensorimotor neuropathy, distal amyotrophy, and pes cavus without elevated levels of AFP or eye abnormalities [10].

Reviewing the literature, several case series, including the largest cohort of 90 patients from Europe, and others from North Africa, North America, and the Middle East, have been reported [12,18,19,20,21,22,23,24] (Table 3). These studies have shown that most AOA2 patients with the age of onset almost within the second decade demonstrated progressive cerebellar ataxia (94–100%), axonal sensorimotor peripheral neuropathy (78–100%), and elevated AFP levels (67–100%). OMA is present in 0–69% of the case series. Additional signs that may be encountered in AOA2/SCAN2 include pyramidal signs (0–31%), strabismus (11–37%), chorea and/or dystonia, or tremor (9.5–57%) and saccadic ocular pursuit (4.5–100%). Of note, our patient has saccadic ocular pursuit without OMA, which was also found in all cases of the three series reported [12,18,24], but found in only 4.5% of the cohort reported by Anheim et al. [22], suggesting that OMA is not an essential feature of AOA2. There are other less common features of AOA2, including pes cavus, elevated CK, mild hypoalbuminemia, and mild hypercholesterolemia [12,17,18,19,20,21,22,23], and very rarely infertility [25].

In most parts of Europe and North Africa, FRDA is regarded as the most common type of ARCA, with AT being the 2nd and AOA2/SCAN2 the 3rd most common. AOA2/SCAN2 has been suspected to be the second most frequent cause of ARCA in adults, after FRDA [11]. The higher prevalence in adults is probably due to the fact that it has a milder course of disease compared with AT (median survival is calculated to be 25 years [26]), hence the higher survival rates in the adult population. Despite being one of the most common ARCAs, AOA2/SCAN2 is still a relatively rare disease with prevalence rates estimated from 1/150,000 [27] to 1/900,000 [11], depending on the region. As shown in Table 4, in East Asian countries, only 11 cases of AOA2 (including the present case) have been reported so far: 8 cases come from Japan [14,28,29,30,31] (population of approx. 126 million) and only 2 cases from China [32] (population of approx. 1.4 billion).

This indicates that the disease prevalence is either much lower in East Asia or it is a result of underdiagnosis. In Taiwan (population of approx. 23 million), there are currently no reported cases of FRDA, AOA1, AOA2/SCAN2, AT, SCAN1, or ATLD. We report here the first case of AOA2 in Taiwan. All cases from Eastern Asian had cerebellar ataxia and polyneuropathy and their age of onset were all within the second decades and 91% of them had elevated AFP, which is similar to series cases reported from Europe, North Africa, North America, and the Middle East. Among the cases from East Asia, 90% had saccadic pursuit and 100% had nystagmus, but none of them had OMA. The results suggest that OMA is rare but saccadic ocular pursuit and nystagmus are common in East Asian AOA2.

### 4.2. NGS and Mutation Interpretation

Due to the heterogeneous presentation of ARCAs and the relative unavailability of genetic testing until recent years, underdiagnoses could be possible. As with our case, the definitive diagnosis was only conclusive after the application of NGS, seven years after the first clinical evaluation. A study using heterogeneous ataxias as a neurogenetic disorder, to assess the introduction of NGS into clinical practice, found that the most likely predictors of detecting a mutation were an adolescent age at onset age, a distinct complex phenotype, a family history, and a progressive disorder [33]. In concordance with this statement, we were able to detect the underlying mutations responsible for our patient’s disease, specifically a compound heterozygous mutation of SETX c.6859C > T (p.R2287X) and c.7034-7036del. The challenge after detecting the mutation was pathogenicity interpretation. In addition to ANNOVAR for annotation of the variants, we further used the database VarCards (http://varcards.biols.ac.cn) (accessed on 5 January 2022) for the c.6859C > T (p.R2287X) mutation (Table 1), and Mutation Taster for c.7034-7036del to further confirm the pathogenicity of these two mutations (Table 2). The results indicate that both mutations have a “damaging effect”.

### 4.3. Genotype/Phenotype

As recorded on The Human Gene Mutation Database (HGMD), so far over 100 mutations have been reported in the SETX gene, most of them being missense/nonsense mutations, followed by small deletions. Among these mutations, there is a maximum ratio in exon 10 of SETX. Genotype/phenotype correlation in AOA2 has not been clearly established, although Anheim et al. [22] found that missense mutations in the C-terminal helicase domain of SETX was associated with a less severe AOA2/SCAN2 phenotype in comparison to those out of the helicase domain or those caused by deletions and truncating mutations of SETX. The mutations in our case, c.6859C > T (p.R2287X) in exon 22 and c.7034-7036del in exon 23, are both located within the C-terminal helicase domain. However, they are not missense mutations. The c.6859C > T (p.R2287X) results in a stop-gain (nonsense) mutation, which leads to a truncated protein, while c.7034-7036del is a deletion type of mutation that causes the loss of an amino acid.

The c.6859C > T (p.R2287X) mutation was previously reported only in two female siblings from Saudi Arabia [8]. Clinical features of the two siblings were teenage onset of ataxia, axonal polyneuropathy, reduced deep tendon reflexes, normal cognitive function, and elevated levels of AFP. Interestingly, the major difference was actually found between the two siblings, presence of OMA, dysarthria, and severe ataxia in the older sibling, but mild ataxia without OMA or dysarthria in the younger sibling (similar to our patient), which suggest the same mutation causes variant phenotypes within the same family. We consider our case as a milder phenotype of AOA2/SCAN2, given that she can still walk, albeit unsteadily without being confined to a wheelchair (disease duration of 13 years). Whether the milder phenotype is related to the mutation type or location remains a debate, and requires further investigation.

Mutations in the SETX gene is associated not only with the disease phenotype of AOA2/SCAN2 (OMIM #606002), which is autosomal recessive, but can also cause autosomal dominant phenotypes, including amyotrophic lateral sclerosis 4 [6], tremor ataxia syndrome [34], and autosomal dominant proximal spinal muscular atrophy [35]. These reports suggest there is no certain genotype–phenotype correlation for SETX mutations.

Although knowledge about the function of SETX has significantly increased in recent years, the exact mechanisms that lead to cerebellar damage and neurodegeneration in AOA2/SCAN2 has yet to be established.

## 5. Conclusions

With NGS application, we identified a novel heterozygous mutation of SETX in a case of ARCA with polyneuropathy and elevated AFP without OMA in Taiwan. The literature review showed that ataxia, polyneuropathy, and elevated AFP are common features and OMA is a variable sign of AOA2 from different populations, whereas OMA is rare and saccadic pursuit and nystagmus are common in East Asian AOA2. So far, there is no effective treatment for AOA2, whereas, with advanced technology and ongoing efforts in research, the chances of disease-modifying therapies in the future may be possible. We hope that by presenting this report, we help facilitate the accurate diagnosis of AOA2/SCAN2 at an early disease stage, in order to improve clinical management and genetic counseling; furthermore, we hope our findings to contribute to further research on the topic.

## Figures and Tables

**Table 1:** The results of predicting the impact of the c.6859C > T:p.R2287X mutation on the structure and function of SETX using the VarCard database. This variant is predicted to be damaging or pathogenic by different algorithms.

Location	Ref	Alt	Gene	Effect	Mutation Type
chr9:135152523-135152523	G	A	ABCD1	Stop-gain	SNV
Amino acids change	SETX:NM_015046:exon22:c.6859C > T:p.R2287X				
Supporting deleterious algorithm	11				
D:A algorithms	11:12				
Damaging score	1.00				
Extreme	Y				
Cytoband	9q34.13				
In silico missense prediction					
Algorithm	Score			Predication	
LRT	0.000			Deleterious	
MutationTaster	1			Disease_causing_automatic	
CADD	48			Damaging	
DANN	0.996			Damaging	
FATHMM_MKL	0.958			Damaging	
Eigen	0.509			Damaging	
GenoCanyon	1.000			Damaging	
fitCons	0.707			Damaging	
GERP++	6.17			Conserved	
phyloP	2.599			Conserved	
phastCons	0.996			Nonconserved	
SiPhy	15.248			Conserved	
ReVe	0.717			Damaging	
Allele frequency in population					
Dataset	Population			Allele frequency	
gnomAD_exome	ALL			4.074 × 10^−^^6^	
gnomAD_exome	African American			0	
gnomAD_exome	Latino			0	
gnomAD_exome	Ashkenazi Jewish			0	
gnomAD_exome	East Asian			0	
gnomAD_exome	Finnish			4.614 × 10^−^^5^	
gnomAD_exome	Non-Finnish European			0	
gnomAD_exome	Other			0	
gnomAD_exome	South Asian			0	
Disease-related information					
Datebase	Information				
InterVar	Likely pathogenic				
InterPro	P-loop containing nucleoside triphosphate hydrolase				

**Table 2:** The results of predicting the impact of the c.7034-7036del mutation on the structure and function of SETX using the Mutation Taster database. This variant is predicted to be disease causing.

Alteration	Chr9:135150707_135150709delTTA		
HGNC symbol	SETX		
Ensembl transcript	ENST00000224140		
GenBank transcript ID	NM_015046		
UniProt peptide	Q7Z333		
Alteration type	deletion		
Alteration region	CDS		
DNA changes	c.7034_7036delTAA cDNA.7217_7219delTAA g.79664_79666delTAA		
AA changes	Deletion of 1 or 2 AA		
Position of altered AA	2344		
Frameshift	no		
Known variant	Variant was neither found in ExAC nor 1000 G		
Regulatory features	H3K36me3, histone, histone 3 lysine 36 Tri-methylation		
phyloP/phastCons		phyloP	phastCons
	(flanking)	4.56	1
		3.785	1
		0.563	1
		3.785	1
	(flanking)	3.785	1
Conservation Protein level for non-synonymous changes	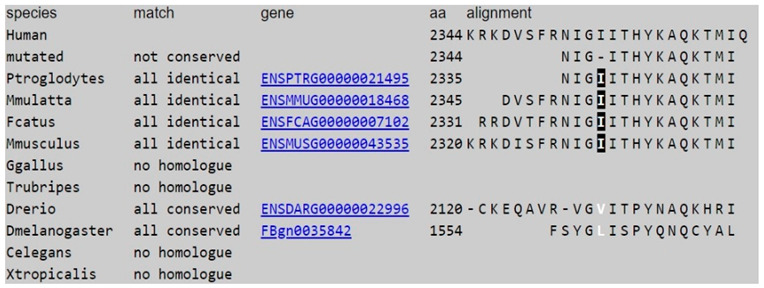		

**Table 3:** Published AOA2/SCAN2 case series from Europe, North Africa, North America, and the Middle East.

AOA2 Series	Duquette et al. [18]	Le Ber et al. [19]	Criscuolo et al. [20]	Tazir et al. [21]	Anheim et al. [22]	Hammer et al. [23]	Nanetti et al. [24]	Mariani et al. [12]
No. of patients	24	18	10	19	90	13	13	11
Age of onset ± SD (mean, range)	14.8 ± NA (2–20)	15 ± 4 (9–25)	20.3 ± NA (3–30)	14 ± 3 (8–19)	14.6 ± 3.4 (7–25)	15.5 ± 3.3 (10–20)	15.6 ± 2.4 (11–18)	12 ± N/A
Cerebellar ataxia	100%	100%	100%	94%	100%	100%	100%	100%
Neuropathy	96%	78%	100%	90%	97.5%	100% in all data available (12/13)	100%	90% in all data available (9/10)
Elevated AFP (>7 ng/mL)	100%	100%	67%	100%	99%	100% in all data available (10/13)	100%	90% in all data available (9/10)
OMA	0%	56%	20%	32%	51%	69%	0%	NA
Pyramidal signs	0%	17%	0%	11%	20.5%	31%	15%	18.2%
Strabismus	29%	11%	NA	37%	12.3%	NA	31%	NA
Chorea and/or dystonia, or tremor	57%	44%	20%	32%	37%	NA	NA	45.5%
Saccadic pursuit without OMA	100%	NA	NA	NA	4.5%	NA	100%	100%

AFP: alpha fetoprotein; OMA: ocular motor apraxia; NA: not available.

**Table 4:** Published AOA2/SCAN2 cases in East Asia.

AOA2 in Eastern Asia	2006 Japan [28]	2009 Japan [29]	2013 Japan [15]	2016 Japan [30]	2018 Japan [31]	2017 China [32]	2021 Taiwan #
No. of patients	2	1	3	1	1	2	1
Age of onset	16, late-teens	17	15, 17, 18	13	NA	16, 20	16
Cerebellar ataxia	2/2	1/1	3/3	1/1	1/1	2/2	1/1
Neuropathy	2/2	1/1	3/3	1/1	1/1	2/2	1/1
Elevated AFP (>7 ng/mL)	2/2	1/1	1/3	1/1	1/1	2/2	1/1
OMA	0/2	0/1	0/3	0/1	NA	0/2	0/1
Pyramidal signs	0/2	NA	NA	0/1	NA	2/2	0/1
Strabismus	NA	NA	NA	0/1	NA	2/2	0/1
Chorea and/or dystonia	NA	NA	NA	0/1	NA	NA	1/1
Saccadic pursuit	2/2	1/1	3/3	0/1	NA	1/2	1/1
Horizontal nystagmus	2/2	1/1	3/3	1/1	NA	1/1	1/1

AFP: alpha fetoprotein; OMA: ocular motor apraxia; NA: not available. # The patient in this study.

## Data Availability

All data included in this study are available upon reasonable request by contact with the corresponding author.

## References

[1] Moreira M.-C., Klur S., Watanabe M., Németh A.H., le Ber I., Moniz J.-C., Tranchant C., Aubourg P., Tazir M., Schöls L. (2004). Senataxin, the Ortholog of a Yeast RNA Helicase, Is Mutant in Ataxia-Ocular Apraxia 2. Nat. Genet..

[2] Suraweera A., Becherel O.J., Chen P., Rundle N., Woods R., Nakamura J., Gatei M., Criscuolo C., Filla A., Chessa L. (2007). Senataxin, Defective in Ataxia Oculomotor Apraxia Type 2, Is Involved in the Defense against Oxidative DNA Damage. J. Cell Biol..

[3] Vantaggiato C., Bondioni S., Airoldi G., Bozzato A., Borsani G., Rugarli E.I., Bresolin N., Clementi E., Bassi M.T. (2011). Senataxin Modulates Neurite Growth through Fibroblast Growth Factor 8 Signalling. Brain.

[4] Becherel O.J., Yeo A.J., Stellati A., Heng E.Y.H., Luff J., Suraweera A.M., Woods R., Fleming J., Carrie D., McKinney K. (2013). Senataxin Plays an Essential Role with DNA Damage Response Proteins in Meiotic Recombination and Gene Silencing. PLoS Genet..

[5] Richard P., Feng S., Tsai Y.-L., Li W., Rinchetti P., Muhith U., Irizarry-Cole J., Stolz K., Sanz L.A., Hartono S. (2021). SETX (Senataxin), the Helicase Mutated in AOA2 and ALS4, Functions in Autophagy Regulation. Autophagy.

[6] Chen Y.-Z., Bennett C.L., Huynh H.M., Blair I.P., Puls I., Irobi J., Dierick I., Abel A., Kennerson M.L., Rabin B.A. (2004). DNA/RNA Helicase Gene Mutations in a Form of Juvenile Amyotrophic Lateral Sclerosis (ALS4). Am. J. Hum. Genet..

[7] Beaudin M., Klein C.J., Rouleau G.A., Dupré N. (2017). Systematic Review of Autosomal Recessive Ataxias and Proposal for a Classification. Cerebellum Ataxias.

[8] Bohlega S.A., Shinwari J.M., al Sharif L.J., Khalil D.S., Alkhairallah T.S., al Tassan N.A. (2011). Clinical and Molecular Characterization of Ataxia with Oculomotor Apraxia Patients in Saudi Arabia. BMC Med. Genet..

[9] Richards S., Aziz N., Bale S., Bick D., Das S., Gastier-Foster J., Grody W.W., Hegde M., Lyon E., Spector E. (2015). Standards and Guidelines for the Interpretation of Sequence Variants: A Joint Consensus Recommendation of the American College of Medical Genetics and Genomics and the Association for Molecular Pathology. Genet. Med..

[10] Fogel B.L., Perlman S. (2007). Clinical Features and Molecular Genetics of Autosomal Recessive Cerebellar Ataxias. Lancet Neurol..

[11] Anheim M., Fleury M., Monga B., Laugel V., Chaigne D., Rodier G., Ginglinger E., Boulay C., Courtois S., Drouot N. (2010). Epidemiological, Clinical, Paraclinical and Molecular Study of a Cohort of 102 Patients Affected with Autosomal Recessive Progressive Cerebellar Ataxia from Alsace, Eastern France: Implications for Clinical Management. Neurogenetics.

[12] Mariani L.L., Rivaud-Péchoux S., Charles P., Ewenczyk C., Meneret A., Monga B.B., Fleury M.-C., Hainque E., Maisonobe T., Degos B. (2017). Comparing Ataxias with Oculomotor Apraxia: A Multimodal Study of AOA1, AOA2 and AT Focusing on Video-Oculography and Alpha-Fetoprotein. Sci. Rep..

[13] Renaud M., Tranchant C., Koenig M., Anheim M. (2020). Autosomal Recessive Cerebellar Ataxias with Elevated Alpha-Fetoprotein: Uncommon Diseases, Common Biomarker. Mov. Disord..

[14] Ichikawa Y., Ishiura H., Mitsui J., Takahashi Y., Kobayashi S., Takuma H., Kanazawa I., Doi K., Yoshimura J., Morishita S. (2013). Exome Analysis Reveals a Japanese Family with Spinocerebellar Ataxia, Autosomal Recessive 1. J. Neurol. Sci..

[15] Lupica A., Di Stefano V., Gagliardo A., Iacono S., Pignolo A., Ferlisi S., Torrente A., Pagano S., Gangitano M., Brighina F. (2021). Inherited Neuromuscular Disorders: Which Role for Serum Biomarkers?. Brain Sci..

[16] Choudry T.N., Hilton-Jones D., Lennox G., Houlden H. (2018). Ataxia with Oculomotor Apraxia Type 2: An Evolving Axonal Neuropathy. Pract. Neurol..

[17] Izatt L., Németh A.H., Meesaq A., Mills K.R., Taylor A.M.R., Shaw C.E. (2004). Autosomal Recessive Spinocerebellar Ataxia and Peripheral Neuropathy with Raised Alpha-Fetoprotein. J. Neurol..

[18] Duquette A., Roddier K., McNabb-Baltar J., Gosselin I., St-Denis A., Dicaire M.-J., Loisel L., Labuda D., Marchand L., Mathieu J. (2005). Mutations in Senataxin Responsible for Quebec Cluster of Ataxia with Neuropathy. Ann. Neurol..

[19] le Ber I., Bouslam N., Rivaud-Péchoux S., Guimarães J., Benomar A., Chamayou C., Goizet C., Moreira M.-C., Klur S., Yahyaoui M. (2004). Frequency and Phenotypic Spectrum of Ataxia with Oculomotor Apraxia 2: A Clinical and Genetic Study in 18 Patients. Brain.

[20] Criscuolo C., Chessa L., di Giandomenico S., Mancini P., Saccà F., Grieco G.S., Piane M., Barbieri F., de Michele G., Banfi S. (2006). Ataxia with Oculomotor Apraxia Type 2: A Clinical, Pathologic, and Genetic Study. Neurology.

[21] Tazir M., Ali-Pacha L., M’Zahem A., Delaunoy J.P., Fritsch M., Nouioua S., Benhassine T., Assami S., Grid D., Vallat J.M. (2009). Ataxia with Oculomotor Apraxia Type 2: A Clinical and Genetic Study of 19 Patients. J. Neurol. Sci..

[22] Anheim M., Monga B., Fleury M., Charles P., Barbot C., Salih M., Delaunoy J.P., Fritsch M., Arning L., Synofzik M. (2009). Ataxia with Oculomotor Apraxia Type 2: Clinical, Biological and Genotype/Phenotype Correlation Study of a Cohort of 90 Patients. Brain.

[23] Hammer M.B., el Euch-Fayache G., Nehdi H., Saidi D., Nasri A., Nabli F., Bouhlal Y., Maamouri-Hicheri W., Hentati F., Amouri R. (2012). Clinical and Molecular Findings of Ataxia with Oculomotor Apraxia Type 2 (AOA2) in 5 Tunisian Families. Diagn. Mol. Pathol..

[24] Nanetti L., Cavalieri S., Pensato V., Erbetta A., Pareyson D., Panzeri M., Zorzi G., Antozzi C., Moroni I., Gellera C. (2013). SETX Mutations Are a Frequent Genetic Cause of Juvenile and Adult Onset Cerebellar Ataxia with Neuropathy and Elevated Serum Alpha-Fetoprotein. Orphanet J. Rare Dis..

[25] Becherel O.J., Fogel B.L., Zeitlin S.I., Samaratunga H., Greaney J., Homer H., Lavin M.F. (2019). Disruption of Spermatogenesis and Infertility in Ataxia with Oculomotor Apraxia Type 2 (AOA2). Cerebellum.

[26] Crawford T.O., Skolasky R.L., Fernandez R., Rosquist K.J., Lederman H.M. (2006). Survival Probability in Ataxia Telangiectasia. Arch. Dis. Child..

[27] Nicolaou P., Georghiou A., Votsi C., Middleton L.T., Zamba-Papanicolaou E., Christodoulou K. (2008). A Novel c.5308_5311delGAGA Mutation in Senataxin in a Cypriot Family with an Autosomal Recessive Cerebellar Ataxia. BMC Med. Genet..

[28] Asaka T., Yokoji H., Ito J., Yamaguchi K., Matsushima A. (2006). Autosomal Recessive Ataxia with Peripheral Neuropathy and Elevated AFP: Novel Mutations in SETX. Neurology.

[29] Nakamura K., Yoshida K., Makishita H., Kitamura E., Hashimoto S., Ikeda S.-I. (2009). A Novel Nonsense Mutation in a Japanese Family with Ataxia with Oculomotor Apraxia Type 2 (AOA2). J. Hum. Genet..

[30] Motokura E., Yamashita T., Takahashi Y., Tsunoda K., Sato K., Takemoto M., Hishikawa N., Ohta Y., Hashiguchi A., Takashima H. (2017). An AOA2 Patient with a Novel Compound Heterozygous SETX Frame Shift Mutations. J. Neurol. Sci..

[31] Kashimada A., Hasegawa S., Nomura T., Shiraku H., Moriyama K., Suzuki T., Nakajima K., Mizuno T., Imai K., Sugawara Y. (2019). Genetic Analysis of Undiagnosed Ataxia-Telangiectasia-like Disorders. Brain Dev..

[32] Lu C., Zheng Y.-C., Dong Y., Li H.-F. (2016). Identification of Novel Senataxin Mutations in Chinese Patients with Autosomal Recessive Cerebellar Ataxias by Targeted Next-Generation Sequencing. BMC Neurol..

[33] Németh A.H., Kwasniewska A.C., Lise S., Parolin Schnekenberg R., Becker E.B.E., Bera K.D., Shanks M.E., Gregory L., Buck D., Zameel Cader M. (2013). Next Generation Sequencing for Molecular Diagnosis of Neurological Disorders Using Ataxias as a Model. Brain.

[34] Bassuk A.G., Chen Y.Z., Batish S.D., Nagan N., Opal P., Chance P.F., Bennett C.L. (2007). In Cis Autosomal Dominant Mutation of Senataxin Associated with Tremor/Ataxia Syndrome. Neurogenetics.

[35] Rudnik-Schöneborn S., Arning L., Epplen J.T., Zerres K. (2012). SETX Gene Mutation in a Family Diagnosed Autosomal Dominant Proximal Spinal Muscular Atrophy. Neuromuscul. Disord..

